# Best practices in developing a national palliative care policy in resource limited settings: lessons from five African countries

**DOI:** 10.3332/ecancer.2016.652

**Published:** 2016-07-07

**Authors:** Emmanuel BK Luyirika, Eve Namisango, Eunice Garanganga, Lidia Monjane, Ntombi Ginindza, Gugulethu Madonsela, Fatia Kiyange

**Affiliations:** 1 African Palliative Care Association, PO Box 72518, Plot 95, Dr Gibbons Road, Kampala, Uganda; 2 Hospice and Palliative Care Association of Zimbabwe, 13 Lezard Avenue, Milton Park, Harare, Zimbabwe; 3 Mozambique Palliative Care Association, Av Eduardo Mondlane NR 540, BR Polana 0101, Maputo, Mozambique; 4 Swaziland National AIDS Programme, Ministry of Health, PO Box 1119, Mbabane, H100, Swaziland

**Keywords:** policy makers, palliative care, palliative care policy, policy development, policy goals, health systems, situational analysis, terms of reference, evidence, government, pain medications, controlled substances, financial resources, stakeholders, consultation

## Abstract

Given the high unmet need for palliative care in Africa and other resource limited settings, it is important that countries embrace the public health approach to increasing access through its integration within existing healthcare systems. To give this approach a strong foundation that would ensure sustainability, the World Health Organisation urges member states to ensure that policy environments are suitable for this intervention. The development, strengthening, and implementation of national palliative care policies is a priority. Given the lack of a critical mass of palliative care professionals in the region and deficiency in documenting and sharing best practices as part of information critical for regional development, policy development becomes a complex process. This article shares experiences with regard to best practices when advocating the national palliative care policies. It also tells about policy development process, the important considerations, and cites examples of policy content outlines in Africa.

## Background

There is an increase in the need for palliative care in Africa, given the increasing disease burden from both communicable and non-communicable diseases [1]. Access to palliative care in the region remains largely sparse despite the high disease burden and particularly diagnoses that can benefit from palliative care [2, 3]. In the majority of African countries, most provision for palliation is from isolated centres of excellence without comprehensive integration into the different levels of the health system structure using palliative care teams as recommended by the World Health Organisation (WHO) [4], and this arrangement cannot support equitable access to all those in need.

The WHO now recommends public health strategies by the way of increasing access to palliative care through integration of palliative care into existing health care systems [5]. It is argued that the public health approach is associated with more cost effective interventions that reach more people and which is more likely to enhance translation of new knowledge into evidence-based interventions suitable for the different contexts and with sufficient buy in from governments and communities [5]. In recent times many countries have been working hard to respond to the need for palliative care which is currently provided using different models ranging from a facility-based hospice to community-based services with home care.

At the launch of the public health approach to palliative care, the WHO also recommended the establishment of policies and programmes that would guide cancer relief and palliative care [6, 7]. Policies play an important role in providing guidance to the palliative care development process and entrenching sustainability of the service. Key stakeholders and experts in the region and from elsewhere have also emphasised the need to develop palliative care policies to guide the palliative care development process in turn wanting greater investment by governments. This is meant to help ensure that the pivotal national changes aimed at improving access to quality palliative care are accommodated within an enabling policy environment. To date six African countries have stand alone national palliative care policies and these are: Malawi, Mozambique, Rwanda, Swaziland, Tanzania, and Zimbabwe. Botswana and Uganda have drafted national policies that is in the process of being adopted. Development partners such as: the American Cancer Society; Open Society Foundations (OSF) in New York; IntraHealth International HIV/AIDS Clinical Services Programme (HCSP) in Rwanda, Open Society Initiative for Southern Africa (OSIEA); United States Agency for International Development (USAID)/Regional HIV/AIDS Programme; USAID Malawi; USAID Tanzania; USAID Rwanda have been instrumental in supporting the development of national palliative care policies in these countries.

In the long term, palliative care services should be integrated into service delivery, especially at primary and secondary level care in order to achieve maximum coverage [8]. It is also critical to ensure that there is appropriate knowledge, skills, and attitudes among all service providers, including care providers, patients, families, and the general public [5].

As countries in Africa take on the policy development challenge, it is important to build their capacity for policy development and also offer guidance and technical assistance using international and regional policy and legislative frameworks. It is important to ensure that policies are evidence based, aligned with national laws and policy development requirements, involve all stakeholders concerned, engage in effective policy dialogues, and ensure that they are backed with carefully planned monitoring and evaluation frameworks. This article presents experiences and best practices on palliative care policy development, lessons, key operational definitions, and justification for policy guidance that can be used for advocacy. All these can be adapted and used within the region.

## Palliative care situation before national palliative care policy adoption and implementation

In most of the African countries palliative care is a new discipline and service despite the huge burden of disease for which palliative care is needed especially because of human immunodeficiency virus (HIV), cancer, and other life-limiting illnesses. The HIV figures for each of the countries in this paper as well as the stages of palliative care development at the time are indicated in the table and in the appendix.

## What is a public policy?

A policy is a public document, thus known as a public policy which shows what the government intends to do or not to do. Public policies are declarations of intent, a programme of goals, and general rules covering future behaviour to important government decisions, a selected line or course of action, the consequences of action or inaction, and even all government action [9]. Policies are broad guidelines for decision making that once established serve as a strategic link between the formulation and implementation of any process or desired action.

**A palliative care policy** is therefore an intention and broad guideline which elaborates government’s goals and actions when it comes to implementation of palliative care services across all levels of the health system. The policy provides a legal framework within which quality and coordinated palliative care services can be made available and accessible to all people who need them in a country.

## Why should African countries develop and implement palliative care policies?

- Access to palliative care is increasingly recognised as a human right, based on the International Human Right to Health from the International Covenant on Economic, Social, and Cultural Rights (ICESCR) Article 12.1 (1996) [10]. African governments are signatories to this right. A national policy makes it possible for governments to set goals for ensuring its universal access.
- African governments are member states of the WHO and are signatories to the resolutions that support the development of palliative care. These include: the May 2014 World Health Assembly (WHA) resolution on palliative care; the December 2012 resolution on Universal Health Coverage, and the November 2011 United Nations political declaration on Non-Communicable Diseases (NCDs). These resolutions urge governments to develop and implement evidence-based national policies on palliative care.
- In 2012, the African Union (AU) adopted a common position on controlled substances and access to pain medications [11] where it urges AU member states to ensure a functioning system for managing the availability of narcotic medicines and psychotropic substances to provide relief from pain and suffering by ensuring the safe delivery of the best affordable medicines to those patients who need them, and at the same time to prevent the diversion of medicines for the purpose of abuse.
- The high and growing burden of diseases coupled with an interplay between communicable and non-communicable diseases.
- The lack of a policy framework is a limitation to government investment in the development of palliative care services, leading to limited access, poor coordination, and inequities in service provision as well as inability to establish public private partnerships in making services more available.
- To consolidate already existing initiatives on hospice and palliative care and provide them with a legal framework.
- This National Palliative Care policy is critical in facilitating the scale-up of palliative care provision that encompasses the relief of pain and other distressing symptoms, right from diagnosis, treatment, end of life care, and bereavement support for families, at all levels of the health system in any country through the adoption of a primary health approach.
- The policy is necessary in providing a framework for standardised implementation of palliative care services through a range of activities undertaken by different stakeholders throughout the country. It provides a one point reference for implementation of palliative care interventions, promotes integrated approaches, as well as sustainability of services.

## What steps can be taken in developing a national palliative care policy?

- The ministry of health and key pioneers develop the **terms of reference** for a palliative care technical working group.
- Forming a policy development technical **working group** that has representation from key stakeholders of palliative care. Such a group may already be in existence, such as a national palliative care working group or task force chaired and hosted by the ministry of health. Missing key players can be co-opted to this group to support the policy development process with special attention to representation from clinical palliative care specialists where they exist, key service providers, academic/training institutions for health and allied workers, legal experts, narcotics experts, cancer and other disease survivors, the national medicines authority, oncology and radio-oncology experts, social workers, registrars of medical, dental, nursing, pharmacy and allied health councils, and other appropriate resource persons.
- Performing a **national situation analysis**. Some countries have identified the need for a national palliative care policy through undertaking a national situation analysis for palliative care. It is through this that the strength, weaknesses, opportunities, and threats for access to hospice and palliative care services are identified to inform policy goals, priorities, and strategies.
- Identifying and documenting the **evidence** that supports the need for a national palliative care policy. This evidence is presented to the ministry of health and other stakeholders in form of an evidence brief. The evidence brief also provides some policy options for consideration by government in the development of a national palliative care policy. This can be combined with the national situational analysis.
- A review of existing health related policies, strategies, plans, and other relevant documents to ascertain what is included on palliative care and gaps which would be addressed by the national palliative care policy.
  This information collected from steps under step to this point can be summarised into an **evidence brief** document please see example via this link: [http://www.who.int/evidence/sure/PCBExecSummarySept2013.pdf]. This evidence brief should informative about policy dialogues among stakeholders.
- A review of existing policies to consider local adaptation rather than re-inventing the wheel could be less time consuming and cost effective.
- Conducting stakeholder **consultation meetings**. These can be done through one to one interviews, focus group discussions, formal meetings, dialogue with national palliative care working groups or task forces, and other approaches including self-administered questionnaires or telephone interviews. The latter two require extensive follow-up to obtain the input of stakeholders.
- Through stakeholder consultation, the policy is reviewed and revised.
- Presentation of the palliative care policy to all levels of required government approval.
- Editing, designing, and policy dissemination that includes national launch if resources are available.

## Important considerations when developing a national palliative care policy.

- Ensure that the policy is brief, to the point, and spells out the role of each stakeholder whether governmental or non-governmental. Policy makers are more likely to read and understand brief documents compared to detailed big documents.
- Remember that the policy can be further elaborated in the implementation guidelines or strategy, a document that is critical to exist along with the national palliative care policy.
- For most of the African governments, the policy must be costed before presenting it to parliament and cabinet. Policy makers always want to know the financial implication of implementing a new policy.
- Always ensure that the Ministry of Health is taking a lead in policy development and more specifically the policy and planning unit of the ministry is involved in the process of palliative care policy development for technical guidance.
- Involve all relevant stakeholders in the process of developing a national palliative care policy. These may include: relevant departments in the Ministry of Health such as the clinical department, non-communicable diseases (NCDs), policy and planning units as well as district or provincial health representatives among others; palliative care advocates, service providers, and educators, palliative care service beneficiaries.
- Identifying key players and potential organisations that may be significant in developing palliative care throughout the country.
- It is always strategic to undertake advocacy and sensitisation activities with members of parliament for their support of the policy once it gets into parliament.
- Transparency in the process of policy development is critical for policy ownership and implementation.

## What resources are needed?

- The policy development process can be costly as it has to be highly participatory, bringing on board all key and potential stakeholders of palliative care in the country.
- Financial resources are required to convene stakeholders meetings as well as conducting the national situational analysis.
- Often financial resources are required for obtaining technical assistance for policy development, including the development of accompanying documents such as costing of the policy, communications strategy, and national strategy for palliative care, and policy implementation plan or framework to operationalise the policy.
- Financial resources to support policy review, editing, printing, launch, and dissemination.
- Material and document resources such as national palliative care policies from other African countries to support any policy adaptations.
- Resource people and institutions who are knowledgeable and experienced in health and/or palliative care policy development.

## Who should be involved in palliative care policy development?

- Policy makers from all relevant government ministries and departments led by the ministries of health. Other ministries include local government, gender and social development, education, labour, and others. Representation of health service beneficiaries including palliative care patients and caregivers; hospice and palliative care providers and all relevant public and private service providers including Oncology, Obstetrics, Paediatrics, and other chronic diseases specific programmes; civil society organisations, academic institutions, and representation of vulnerable groups, public, and private health/medical insurance stakeholders among others.
- International organisations such as the WHO country office; Centres for Disease Control and Prevention (CDC); other UN development agencies; development partners and donors.

## What is included in a national palliative care policy? Palliative care policy framework

The national situation analysis and consequently national palliative care policy should be aligned with the WHO six core components (building blocks) of health systems: (i) leadership/governance; (ii) service delivery; (iii) health workforce; (iv) health information systems; (v) access to essential medicines, and (vi) financing [12].

These should also be aligned with the WHO public health strategy for effectively integrating palliative care into a country’s system which addresses four key pillars: (i) appropriate policies; (ii) adequate drug availability; (iii) education of policy makers, health care workers and the public, and (iv) implementation of palliative care services at all levels throughout the society [5].

In addition the policy framers should ensure that all the nine responsibilities and obligations of WHO member states as elaborated in the 2014 World Health Assembly Palliative Care Resolution [13] to which all member states committed are adequately covered in the policy. The goal of the resolution is to ensure the strengthening of palliative care as a component of comprehensive care throughout the life course.

Every country has a standard way of presenting its national policies. Therefore, a national palliative care policy must follow the national approach and format for policy development. Table 1 shows examples of content of some of the existing palliative care policies in a few African countries.

## Conclusion

There are several lessons learnt from each of the countries where a national palliative care policy has been developed.

Firstly, there should be assessments performed prior to policy development to help countries identify key gaps that need to be covered and addressed by policy implementation. This will include models for palliative care delivery that need strengthening as well as training and human resource needs.

Secondly, palliative care policy development commits governments to bring back palliative care both as a discipline and as a service package of health care that it must deliver. This is happening in a situation where much of palliative care has been provided by NGOs. In addition this becomes a first step for government to start committing resources, however, little to palliative care delivery.

Thirdly, this makes palliative care advocacy easier across government departments, NGO, and private sector players within these countries when and if there is a national document that commits government.

Fourthly, policies act as a reminder for government to deliver on their regional and global commitments such as the African Common Position on pain medications and controlled substances of 2012 as well as the World Health Assembly Palliative Care resolution of 2014.

At a regional level, when some countries adopt their palliative care policies it becomes easier to engage other countries without having to support them to develop theirs.

Finally, the presence of national palliative care policies helps the other regional and global entities to create awareness about palliative care and also lobby for more funding for palliative care.

Additional resources can be found on the website http://www.who.int/evidence/sure/guides/en/.

## Conflict of interest

The authors declare that they have no conflict of interest.

## Figures and Tables

**Table 1.** A framework with examples of content in national palliative care policies in Africa.

This framework provides content outlines of some of the approved and national hospice and palliative care policies in Africa as of May 2016				
**Country**	**Disease burden in the country and palliative care rating**	**Title and status of policy**	**Content outline**	**Some areas of impact of national palliative care policies**
1. Mozambique	HIV Prevalence (UNAIDS 2012): 11.1% People living with HIV (UNAIDS 2012): 1,600,000 AIDS deaths (UNAIDS 2012): 77,000 WHO Global Palliative Care Atlas 2014 rating of palliative care development: Group 3a with isolated provision up from 2a with just capacity building in previous rating	**National Palliative Care Policy, July 2012 (*approved*)**	1. INTRODUCTION 1.1. Contextualisation 1.2. Context at the African Context 2. VISION, MISSION, and GOALS 2.1. Vision 2.2. Mission 2.3. Goals 3. GUIDING PRINCIPLES 3.1. Encourage adequate coordination and organisation of palliative care in the integrated care system 3.2. Develop and create human resources capacity 3.3. Promote work in multidisciplinary networks on care matters and stimulate inter-sectoral actions 3.4. Promote social and community participation to encourage interaction with the Health Unit 3.5. Holistic assistance to patients focused on their quality of life (comfort and reassurance of the patients) 3.6. Development of research to mobilise the practice of PC 4. POLICY: RESPONSIBILITY FOR MANAGEMENT 5. SPECIFIC ACTIONS 6. MONITORING AND EVALUATION 7. BIBLIOGRAPHY ANNEXES Annex 1: DEFINITION OF CONCEPTS	1. The country has identified funding gaps for palliative care implementation and needs support. 2. The country has developed a model of implementation of palliative care at district level. 3. A local oral morphine reconstitution plan is being developed with support from APCA with support from the American Cancer Society.
2. Swaziland	HIV Prevalence (UNAIDS 2012): 26.5% People living with HIV (UNAIDS 2012): 210,000 AIDS deaths (UNAIDS 2012): 5,500 WHO Global Palliative Care Atlas 2014 rating of palliative Care development: Group 3b with generalised provision	Kingdom of Swaziland, Ministry of Health, National Palliative Care Policy, November 2011 (approved)	1. INTRODUCTION 1.1. Background to Palliative Care 1.2. Swaziland’s experience in the provision of palliative care 1.3. Opportunities 1.4. Policy statement 1.5. Policy Environment 2. VISION AND MISSION STATEMENTS 3. JUSTIFICATION OF THE PALLIATIVE CARE POLICY 3.1. Rationale for policy 4. GOALS AND OBJECTIVES OF PALLIATIVE CARE POLICY 4.1. Overall goal 4.2. Objectives 5. GUIDING PRINCIPLES OF PALLIATIVE CARE 5.1. Human right based approach 5.2. Multisectoral approach 5.3. Quality assurance 5.4. Meaningful involvement of people living with life-limiting illnesses 5.5. Holistic and comprehensive management 6. POLICY APPLICATION 6.1. Policy Issue 6.2. Key policy Issues 7. POLICY FRAMEWORK 7.1. Policy statements and strategies 7.2. Service delivery including palliative care for carer through decentralisation of palliative care services 7.1.1. Policy statement 7.1.2. strategies 7.2. Service availability, equity, and equality 7.2.1. policy statement 7.2.2. strategies 7.3. Quality improvement and assurance 7.3.1. Policy statement 7.3.2. strategies 7.4. Communication and advocacy 7.4.1. Policy statement 7.4.2. Strategies 7.5. Capacity building into all training sessions 7.5.1. Policy statement 7.5.2. Strategies 7.6. Supervision 7.6.1. Policy statements 7.6.2. Strategies 7.7. Referral system 7.7.1. Policy statement 7.7.2. Strategies 7.8. Monitoring and Evaluation 7.8.1. Policy statement 7.8.2. Strategy 7.9. Coordination of care and services 7.9.1. Policy statement 7.9.2. Strategies 7.10. Institutional framework for implementation 7.10.1. Policy implementation responsibilities 7.10.2. Policy statement 7.10.3. Strategies 8. LEGISLATION 8.1. Policy statement 8.2. Strategies 9. RESOURCE IMPLICATIONS 9.1. Financial resources 9.2. Human and institution resources 9.3 Training and supervisory tools 10. POLICY EVALUATION 11. POLICY REVISION	1. National system now avails reconstituted oral morphine for palliative care. 2. Palliative care training has been embarked on.
3. Zimbabwe	HIV Prevalence (UNAIDS 2012): 14.7% People living with HIV (UNAIDS 2012): 1,400,000 AIDS deaths (UNAIDS 2012): 39,000 WHO Global Palliative care Atlas 2014 rating of palliative care development: group 4a with preliminary integration	The National Palliative Care Policy August 2014 (Approved)	FOREWORD ACKNOWLEDGMENTS ACRONYMS INTRODUCTION • PALLIATIVE CARE CONCEPT • RATIONALE FOR A NATIONAL PALLIATIVE CARE POLICY IN ZIMBABWE • MODELS FOR PALLIATIVE CARE IMPLEMENTATION • BACKGROUND OF PALLIATIVE CARE IN ZIMBABWE • GOAL/PURPOSE OF THE PALLIATIVE CARE POLICY Vision Mission Aims GUIDING PRINCIPLES FOR PALLIATIVE CARE POLICY PRINCIPLE 1: HUMAN RIGHTS • Preamble • Policy statements PRINCIPLE 2: SUSTAINABLE PALLIATIVE CARE • Preamble • Policy statements PRINCIPLE 3: ACCESSIBLE PALLIATIVE CARE • Preamble • Policy statements PRINCIPLE 4: HOLISTIC SERVICES FOR QUALITY OF LIFE • Preamble • Policy statements PRINCIPLE 5: CHILDREN’S PALLIATIVE CARE • Preamble • Policy statements PRINCIPLE 6: EDUCATION, TRAINING, AWARENESS AND SUPPORT FOR PALLIATIVE CARE • Preamble • Policy statements PRINCIPLE 7: RESEARCH AND EVIDENCE BASED PRACTICE • Preamble • Policy statements PRINCIPLE 8: MONITORING AND EVALUATION OF PALLIATIVE CARE • Preamble • Policy statements GLOSSARY REFERENCES APPENDIX	
4. Malawi	HIV Prevalence (UNAIDS 2013): 15.2% People living with HIV (UNAIDS 2012): 1,000,000 AIDS deaths (UNAIDS 2012): 48,000 WHO Global palliative care Atlas 2014 rating of palliative Care development: Group 4a with preliminary integration up from category 3 in previous rating	The national policy was approved in 2014	FOREWORD ACKNOWLEDGMENTS LIST OF ACRONYMS AND ABBREVIATIONS GLOSSARY 1. INTRODUCTION 1.1. Background 1.2. Malawi’s experience in the provision of palliative care 1.2.1. Implementation of palliative care services 1.2.2. Palliative care education 1.2.3. Availability of essential medicines 1.2.4. Appropriate policies 1.3. Rationale for a palliative care policy 1.4. Linkage with other relevant policies 2. VISION AND MISSION 2.1. Vision 2.2. Mission statement 3. BROAD POLICY DIRECTIONS 3.1. Overall policy goal 3.2. Policy outcome 3.3. Policy objectives 3.4. Guiding principles 4. POLICY APPLICATION 4.1. Policy use 4.2. Key policy issues 5. POLICY STATEMENTS 5.1. Equitable access to quality and comprehensive palliative care services 5.2. Access to pain relieving medicines, particularly opioids. 5.3. Capacity building 5.4. Resource allocation 5.5. Information, education, and communication (IEC) 5.6. Patient and family participation 5.7. Research and results dissemination 5.8. Coordination and supervision 5.9. Referral system 5.10. Motivation 5.11. Palliative care for paediatric patients 6. IMPLEMENTATION ARRANGEMENTS 6.1. Ministry of health 6.2. Zonal offices 6.3. Central hospitals 6.4. District health offices: 6.5. Health centres 6.6. Professional regulatory boards 6.7. Central medical stores and facility pharmacies 6.8. Civil society 6.9. Health care training institutions 6.10. Patients, families, and communities 7. IMPLEMENTATION PLAN 8. MONITORING AND EVALUATION 9. POLICY REVIEW	1. National palliative care need estimate has been done. 2. National reporting mechanisms for palliative care is in place at the ministry of health. 3. Government is covering 58% of all palliative care provision while churches and NGOs cover the rest. 4. Local reconstitution of morphine powder into oral morphine is in place. 5. Morphine now available in government and faith based health units. 6. Palliative care training has been boosted and the country has over 300 trainers.
5. Tanzania	HIV Prevalence (UNAIDS 2012): 4.1% People living with HIV (UNAIDS 2012): 1,500,000 AIDS deaths (UNAIDS 2012): 80,000 WHO Global palliative care Atlas 2014 rating of palliative Care development: Group 4a with preliminary integration up from category 3 in previous rating	Approved in 2014	1. INTRODUCTION 2. VISION AND MISSION 3. GOALS AND OBJECTIVES 4. GUIDING PRINCIPLES 5. POLICY APPLICATION AND USE 6. POLICY ISSUES, STATEMENTS, AND STRATEGIES 7. POLICY LEGISLATION 8. RESOURCE MOBILISATION 9. POLICY EVALUATION AND REVISION 10. ROLES AND RESPONSIBILITIES OF KEY ACTORS	1. Expansion of palliative care sites with both government and NGO sites offering. 2. Decentralisation of oral morphine reconstitution to regional centres. 3. Integration of palliative care into teaching of health care workers.
